# Actinium-225-PSMA-617 treatment in a patient with advanced prostate cancer causes secondary myelofibrosis: a case report and literature review

**DOI:** 10.3389/fmed.2025.1569143

**Published:** 2025-07-16

**Authors:** Ziye Wang, Wen Tang, Mingwen Liu, Zhifei Xie, Yi Li, Jiang Du, Tao Wu

**Affiliations:** ^1^Department of Urology, The Affiliated Hospital of Zunyi Medical University, Zunyi, China; ^2^Department of Urology, Kaiyang County People's Hospital, Guiyang, Guizhou, China

**Keywords:** Ac-225-PSMA-617, secondary myelofibrosis, metastatic castration-resistant prostate cancer, radioligand therapy, case report

## Abstract

Advanced prostate cancer (PCa) is still an incurable disease. Still, the field of PSMA-targeted radioligand therapy is developing rapidly and is playing an increasingly important role in the treatment of advanced Pca in the future. As an α -particle emitter, Ac-225 shows a potent killing ability for tumor cells due to its short range and high energy deposition in the tumor micrometastasis focus. However, the secondary myelofibrosis (SMF) associated with Ac-225-PSMA-617 treatment is a significant concern. We present a case of metastatic castration-resistant prostate cancer (mCRPC) who developed pancytopenia following the Ac-225-PSMA-617 treatment period, a bone marrow biopsy confirmed SMF and remained uncorrected after multiple component transfusions and symptomatic supportive therapy. Ac-225-PSMA-617 has demonstrated promising therapeutic efficacy in the management of advanced PCa; however, the potential risks associated with SMF necessitate careful consideration. Through comprehensive analysis of this clinical case and comparative evaluation with the existing literature, this study highlights the need to balance clinical benefit with increased vigilance for treatment-related adverse events.

## Introduction

Prostate cancer (PCa) is one of the most common malignancies in men, and the treatment of advanced PCa is particularly challenging (1). Ac-225-PSMA-617 has shown great advantages in the treatment of advanced PCa due to its potential to induce double-strand breakage, DNA cluster breakage, and cell killing (2). Previous studies have shown that 63% of patients treated with Ac-225-PSMA-617 achieved a decrease in prostate-specific antigen (PSA) of>50% within 8 weeks of treatment, 87% of patients observed a degree of PSA remission, the median tumor control time was 9 months, and 5 patients had sustained remission for more than 2 years. Compared with β emitter 177Lu-PSMA, PSA response rate (63 vs. 30%−59%) and complete remission rate (13 vs. <1%) were significantly improved, especially in refractory patients with high tumor burden (45% of patients showed “hyperimaging” pattern on bone scan) and multiple lines of treatment failure (average of 4 previous treatments) (3).

This case report describes a unique and clinically significant event of secondary myelofibrosis (SMF) following treatment with Ac-225-PSMA-617 in a patient with metastatic castration-resistant prostate cancer (mCRPC). SMF is a rare but serious complication characterized by myelofibrosis (MF), leading to pancytopenia, splenomegaly, and bone marrow failure (4). Although MF has been well documented in hematologic malignancies such as myeloproliferative neoplasms (MPNs), the occurrence of secondary complications as a result of PCa radioisotope therapy has not been widely reported (5). The mechanism of AC-225-induced SMF is not clear and may involve direct bone marrow toxicity, radiation-induced stromal activation, or inflammatory cytokine dysregulation (6–8). Given the increasing use of PSMA-targeted radioligands in advanced prostate cancer, it is critical to understand the rare but serious toxicity. This case highlights the need for long-term safety monitoring and the potential mechanisms of bone marrow dysfunction after radioligand therapy.

## Case presentation

**Figure 1:**
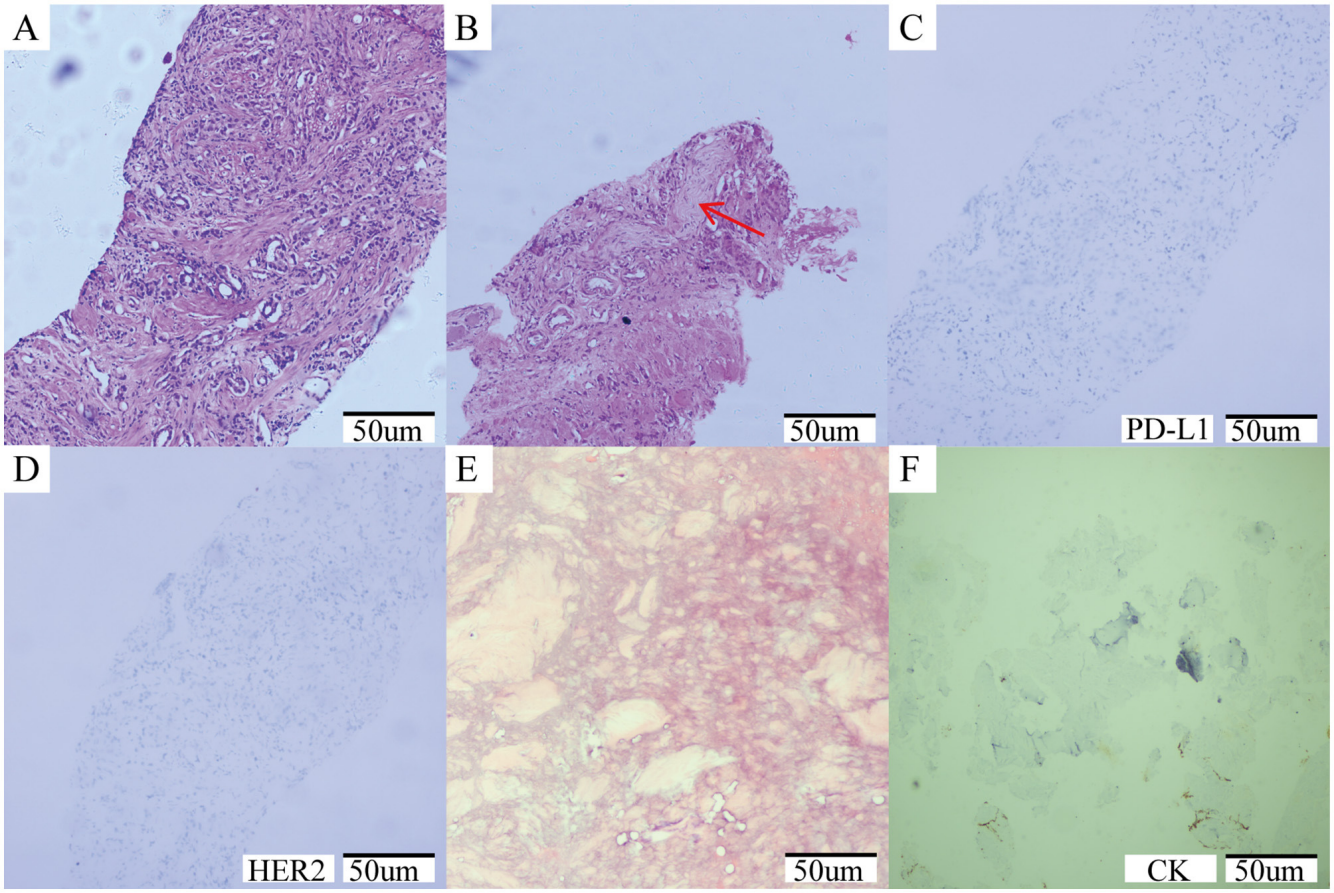
**(A–D)** Prostate needle biopsy histopathology. **(A)** The initial diagnosis of prostate cancer was shown by the puncture image, ISUP grade 3, Gleason score: 4 + 3 = 7; **(B)** Tumor invasion of nerve (red arrow); **(C)** Immunohistochemical results: PDL-1 negative; **(D)** Immunohistochemical results: HER2 negative. **(E, F)** Histopathology of bone marrow biopsy. **(E)** A large number of fibrous tissue proliferation was seen, no hematopoietic cells were seen, no clear cancer; **(F)** Immunohistochemical results: CK negative.

**Figure 2:**
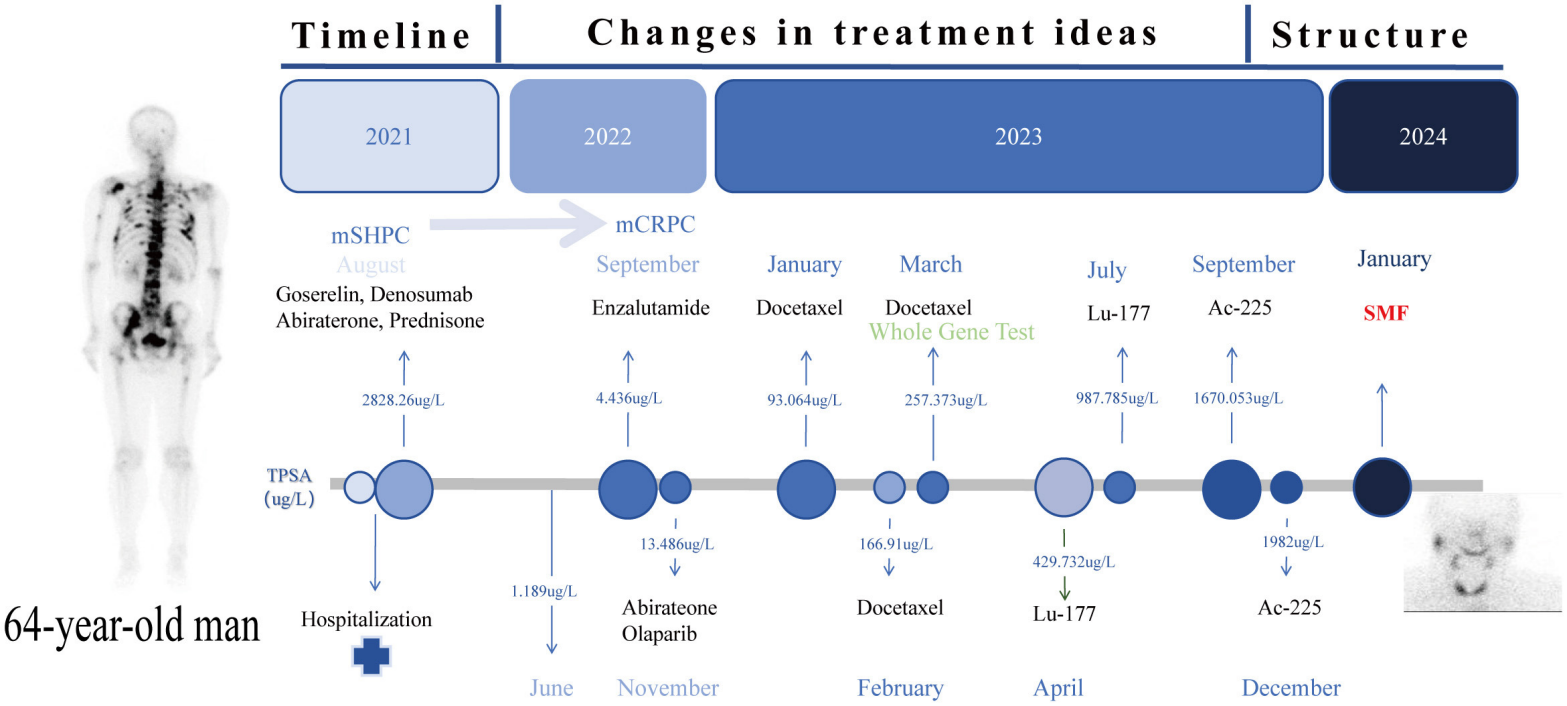
Timeline Roadmap: the time route map of the whole treatment process of the patient and the TPSA changes at key nodes.

A 64-year-old male patient was admitted in August 2021 with “progressive pain in the right hip for 4 months”. The patient had a penicillin allergy with no prior history of myeloid neoplasms, tuberculosis, acquired immunodeficiency syndrome (AIDS), systemic lupus erythematosus (SLE), or other chronic diseases. There was no history of smoking, alcohol consumption, or familial predisposition to malignancies. After admission, laboratory tests showed that the serum total prostate-specific antigen (TPSA) was significantly increased to 2828.26 ng/ml, free PSA (FPSA) was 1387.17 ng/ml, and F/T ratio (% FPSA) was 0.487. Digital rectal examination: hard nodules can be felt, which are harder than normal glands. Prostate MRI plain scan + enhancement (PI-RADS 4 score: T2WI4 score, DWI4 score, DCE4 score): hypointense nodular lesions with ill-defined margins are observed in the mid portions of bilateral peripheral zones and left central zone-transition zone region on T2-weighted imaging. Indistinct delineation of the posterior prostatic capsule. The lesion involved the scrotum. SPECT showed multiple, scattered and irregular abnormal concentration shadows (multiple metastases in lumbar vertebrae, pelvic bones, bilateral femurs, bilateral scapulae, upper parts of bilateral femurs, and thoracic vertebrae). Punch biopsy confirmed PCa (Gleason 4 + 3 = 7), International Society of Urological Pathology (ISUP) grade 3, with neurovascular invasion. Immunohistochemistry showed PD-L1 (-) and HER2 (-) (Figures 1A–D). The patient was diagnosed upon admission with metastatic hormone-sensitive prostate cancer (mHSPC), T4N1M1. We meticulously documented the medications used at each treatment stage and the TPSA values at key milestones to better review the patient's disease progression (Figure 2).

In the mHSPC treatment phase (August 2021-June 2022), the initial treatment regimen was androgen deprivation therapy (ADT) combined with Abirateone. Imaging suggested that tumor progression was controlled (June 2022, TPSA:1.189 ng/ml). After 12 months of treatment (TPSA:4.436 ng/ml September 2022), the disease progressed to mCRPC.

In the mCRPC treatment phase (September 2022–April 2023), we tried Enzalutamide alone, Abirateone combined with Olaparib, and chemotherapy (Docetaxel) combined with ADT, but none of them could control the progression of the tumor, and TPSA increased to 257.373 ng/ml. Genome analysis suggests (Table 1) that a frameshift mutation in exon 16 of APC leads to low responsiveness to enzalutamide and abiraterone; an alternative splicing mutation in exon 8 of AR results in abiraterone resistance; a reduction in PTEN gene copy number leads to abiraterone resistance; homologous recombination repair (HRR) pathway is intact (no interventionable variants). After discussion, it was decided to try radionuclide therapy.

**Table 1:** Key genetic mutations and their clinical implications.

**Gene**	**Alteration**	**Variant type**	**Clinical significance**	**Associated pathway/mechanism**	**Therapeutic implication**
APC	c.4778dupA (p.P1594Afs^*^38)	Frameshift mutation	Class II pathogenic variant; linked to Wnt/β-catenin activation and resistance to AR-targeted therapies	Wnt signaling dysregulation → cancer stem cell expansion	Reduced response to Enzalutamide/Abiraterone
AR	c.2632A>G (p.T878A)	Missense mutation	Class II pathogenic variant; confers agonistic activity to Abiraterone metabolites	Ligand-binding domain alteration → constitutive AR activation	Abiraterone resistance
PTEN	Copy number loss (CN = 1.29)	Somatic deletion	Class II pathogenic variant; drives PI3K/AKT/mTOR hyperactivation	PI3K-AKT pathway activation → tumor proliferation	Resistance to Abiraterone; potential sensitivity to AKT/mTOR inhibitors
HRR gene	(BRCA1\2, FANCA, NBN, EPCAM, MSH6, CHEK2, MSH6, RAD51D)	Germline variants	The clinical significance is unclear or benign	Homologous recombination repair (HRR) proficiency	Excludes PARP inhibitor eligibility (e.g., Olaparib)

Key genetic mutations and therapeutic implications. This table summarizes key mutations found in the patient's genome, highlighting those most relevant to treatment decisions.

APC: Frameshift mutation (exon 16) with 28.02% abundance, classified as pathogenic (Class II). Associated with Wnt-driven stemness and therapy resistance.

AR: T878A mutation (exon 8) promotes ligand-independent activation, a known mechanism of Abiraterone resistance.

PTEN: It is associated with aggressive disease and PI3K pathway dependence. This mutation conferred resistance to abiraterone but sensitivity to AZD8186 and Lpatasertib.

HRR Testing: No actionable germline variants detected. Variants of uncertain significance (e.g., RAD51D, MSH6) classified as benign/likely benign per ACMG guidelines.

During the radionuclide therapy phase (April 2023-December 2023), Ra-223 was not introduced at the hospital where the patient was treated. Therefore, Lu-177-PSMA-617 was tried for a total of 2 cycles (6 GBq/cycle, 8 weeks apart; April and July 2023) in combination with ADT, Enzalutamide, and Denosumab. During treatment with Lu-177-PSMA-617, the patient's bone pain symptoms were relieved and PSA temporarily decreased. However, after 2 cycles, PSMA-PET-CT imaging progressed (September 2023 TPSA:1670.053 ng/ml), and bone pain symptoms were recurrent. In addition to the above symptoms, the patient did not report any other discomfort before or after Lu-177-PSMA-617 treatment. A reexamination of renal function and blood routine showed that they were in a normal state. After 2 months of discontinuation of Lu-177-PSMA-617 treatment, it was decided to switch to Ac-225-PSMA-617 treatment (8 MBq/cycle, 8 weeks apart; September and December 2023). After 2 cycles of AC-225-PSMA-617 treatment, TPSA decreased by about 40% and the pain score improved (NRS 8 → 3). Before the third cycle, the patient complained of fatigue, xerostomia, tasteless eating, increased bone pain symptoms, and SPECT showed multiple tumor bone metastasis and bone destruction. Subsequently, relevant auxiliary examinations were performed: severe adverse reactions were found after Ac-225-PSMA-617 treatment. The patient was defined as having grade 4 thrombocytopenia, grade 4 anemia, grade 2 leukopenia, and grade 2 xerostomia according to the CTCAE v5.0 standard for recording hematological and non-hematological side effects. In addition to pancytopenia, the patient's laboratory test results also indicated elevated alkaline phosphatase and decreased reticulocyte count, with nucleated red blood cells observed in peripheral blood. Bone marrow biopsy results showed extensive fibrous tissue proliferation, no hematopoietic cells, and no clear cancer. Immunohistochemistry results were CK (-) (Figures 1E, F). Previous genetic testing did not detect mutations in JAK2, CALR, MPL, or other genes. After ruling out other possible diseases, the patient was diagnosed with SMF grade 2.

Due to the severe side effects, treatment of PCa had to be stopped and switched to correct the hematological decline: alternating use of recombinant human thrombopoietin (rhTPO) (subcutaneous injection, 15,000 U/day) and eptinezole ethyl alcohol tablet (oral, 50 mg). Each course consisted of rhTPO treatment for 14 days, followed by eptinezole ethyl alcohol tablet for 14 days after rhTPO withdrawal. After a total of 2 months of treatment, the platelet fluctuation was between (10-15) × 10^9^/L, and the treatment effect was not good, so intermittent transfusion of platelets and red blood cells is required to maintain.

After the cessation of radionuclide therapy, we continued to follow up closely. However, the patient developed multiple metastases of the tumor: lung, brain, and meninges. The patient's physical condition deteriorated rapidly. Finally, on May 1, 2024, the patient died of respiratory depression.

## Discussion

We found in the published literature (Table 2) that most studies showed that Ac-225-PSMA-617 had a good safety profile in the treatment of advanced PCa, with rare serious adverse events, even in patients of varying disease severity. The occurrence of SMF after Ac-225-PSMA-617 treatment of advanced prostate cancer has not been systematically reported.

**Table 2:** Safety and efficacy of Ac-225-PSMA-617 in the treatment of metastatic castration-resistant prostate cancer: a summary of key studies.

**Author (Year)**	**Age**	**No. of patients**	**Radiation dose per cycle**	**Diagnosis**	**Adverse effects**	**PFS/OS**	**Secondary myelofibrosis (this case)**
Kratochwil et al. (2018) (3)	70	40	100 kBq/kg	mCRPC (multiple prior treatment failures)	Xerostomia (main side effect), hematologic toxicity	PFS: 7 months, OS: >12 months	No
Khreish et al. (2020) (20)	-	30	5.3 MBq	mCRPC (combined with Lu-177-PSMA)	Xerostomia (mild), hematologic toxicity (manageable)	PFS: 19 weeks, OS: 48 weeks	No
Feuerecker et al. (2021) (21)	72	26	9 MBq	mCRPC (after Lu-177-PSMA failure)	Xerostomia (all patients), hematologic toxicity(Grade ¾)	PFS: 3.5 months, OS: 7.7 months	No
Rosar et al. (2021) (22)	77	15	2.7 ± 1.1 MBq	Highly advanced mCRPC (poor prognosis)	Grade 3 anemia in 2 patients; grade 1–2 xerostomia in 2/15 patients; no severe acute adverse events	PFS: 9.1 months, OS: 14.8 months	No
Lawal et al. (2022) (5)	60–77	106	4–8 MBq	mCRPC (mainly bone metastasis)	One patient had grade 4 thrombocytopenia; Grade 3 anemia (0.9%), leukopenia (2.8%), and thrombocytopenia (1.9%); Other: No serious non-hematologic toxicities were reported	PFS: 14 months, OS: 15 months	No
Satapathy et al. (2022) (15)	76	1	8 MBq	mCRPC	Delayed nephrotoxicity (tubulointerstitial nephritis); Grade 2 xerostomia	Follow-up: 6 months	No
Ballal et al. (2023) (23)	67	56	100–150 kBq/kg	End-stage mCRPC (exhausted standard of care)	70% fatigue (1-2 grade); 32.1% xerostomia (1-2 grade); 3.5% grade 3 anemia; 1.7% grade 3 nephrotoxicity	PFS: 9 months, OS: 15 months	No
Sathekge et al. (2024) (24)	68	488	8 MBq	Advanced mCRPC (≥1 prior therapy)	Xerostomia (main side effect), hematologic toxicity, nephrotoxicity	PFS: 15.5 months, OS: 7.9 months	No

Ac-225-PSMA-617, Actinium-225 Prostate-Specific Membrane Antigen Therapy; PFS, Progression-Free Survival; OS, Overall Survival.

In our case, the patient experienced no adverse reactions during the mHSPC endocrine therapy phase. After progressing to mCRPC, genetic testing indicated that the patient was resistant to multiple drugs. The patient experienced mild hematological toxicity during the third chemotherapy phase, but this was quickly corrected with symptomatic treatment. Hematological toxicity from chemotherapeutic agents primarily manifests as bone marrow suppression, such as leukopenia, anemia, and thrombocytopenia, but these adverse reactions mostly improve with symptomatic treatment (9). Due to chemotherapy's poor efficacy, after the blood routine was normal, radionuclide Lu-177 treatment was initiated, during which no adverse reactions such as hematological toxicity occurred. About 2 months after discontinuing Lu-177-PSMA-617, Ac-225-PSMA-617 treatment was administered. However, after two cycles of Ac-225-PSMA-617 treatment, the patient developed severe adverse reactions.

MF is a chronic myeloproliferative disorder characterized by reactive MF caused by the abnormal secretion of cytokines from hematopoietic stem cells. It is associated with polycythemia vera, idiopathic thrombocytosis, chemotherapy, radiotherapy, multiple myeloma, leukemia, or metastatic diseases. Angiogenesis, bone sclerosis, and extramedullary hematopoiesis are common manifestations of SMF (4). The MF grading criteria in WHO 2016 were: MF-0: no cross-dispersed linear reticular stromal protein, consistent with normal bone marrow; MF-1: numerous cross-dispersed reticular stromal protein networks, especially around blood vessels; MF-2: extensive cross-dispersed and dense reticular stromal protein proliferation, occasionally with focal thick fibrous bundles composed of collagen and/or focal bone sclerosis; MF-3: extensive cross-dispersed and dense reticular stromal protein proliferation, as well as rough thick fibrous bundles consisting of collagen, usually accompanied by bone sclerosis (10). After treatment with Ac-225-PSMA-617, the patient experienced fatigue, and laboratory tests revealed extramedullary hematopoiesis. However, the patient did not have typical symptoms of primary myelofibrosis (PMF; such as splenomegaly, tear-drop erythrocytes), and previous genetic testing results did not identify PMF-related gene mutations such as JAK2, CALR, MPL (10). The patient also had no history of tuberculosis, AIDS, SLE, myeloid tumors, or other diseases. After excluding the possibility of other diseases, a bone marrow biopsy confirmed that the tumor had not metastasized to the bone marrow and also confirmed the occurrence of SMF-2 grade.

The severe adverse reactions caused by radionuclide therapy increase the burden on patients and reduce their quality of life. Compared to β particles, α particles have a shorter range but transfer higher linear energy in tissues, making them more lethal to tumor cells. However, they also cause significant radiation damage to normal tissues, especially bone marrow cells near the tumor or treatment target area (6). The half-life of Ac-225 is approximately 9.92 days (11) and that of Lu-177 is approximately 6.64 days (12). Renal function was reviewed before the use of Ac-225 and no abnormalities were found, that is, when Ac-225 was used, Lu-177 was almost excreted from the body through the kidney (13) Studies have shown that Lu-177 treatment can cause a degree of hematological toxicity, but it is usually mild and reversible (14). In addition, Ac-225 has a certain degree of nephrotoxicity (15), and the accumulation of radionuclides in the kidney and urine may lead to kidney damage, which in turn affects kidney function, including the ability to excrete Ac-225, thus exacerbating the risk of SMF. Therefore, we believe that SMF was caused by Ac-225-PSMA-617 treatment and not by the cumulative toxicity of multiple uses of different nuclides.

We speculate that the mechanism of Ac-225-induced SMF may involve three aspects of synergistic effects: direct bone marrow toxicity, radiation-induced stromal activation, and inflammatory cytokine dysregulation (6–8). First, the high linear energy transfer (LET) α particles released by Ac-225 can directly damage the bone marrow microenvironment by inducing DNA breakage of bone marrow cells, inhibiting hematopoietic stem cell function and abnormal clonal proliferation (such as mutant-driven precursor cell expansion), leading to failure of normal hematopoietic function (6, 16). Secondly, α particle radiation may activate bone marrow stromal cells and trigger SMAD-dependent and non-dependent pathways (such as MAPK/PI3K) by upregulating the TGF-β signaling pathway (7), promoting fibroblast to myofibroblast transformation, excessive deposition of extracellular matrix such as collagen, and inhibiting stromal degradation (17). In addition, the damage-associated molecular patterns (DAMPs) released by tumor cells after being killed by particles can activate macrophages and neutrophils, leading to abnormal upregulation of inflammatory factors such as IL-1β, IL-6, and TNF-α (18). These factors not only directly stimulate fibroblast proliferation but also work in concert with TGF-β to form a positive feedback loop of chronic inflammation-fibrosis, further exacerbating the remodeling of the bone marrow microenvironment. Although Ac-225-PSMA-617 has shown significant efficacy in the treatment of bone metastasis of prostate cancer (19), its multiple fibrogenic mechanisms suggest that dynamic monitoring of inflammatory factors (such as IL-6) and fibrosis markers (such as TGF-β) may be required to optimize the treatment strategy to balance the efficacy and the risk of myelotoxicity.

In summary, this case report highlights the efficacy of Ac-225-PSMA-617 in treating advanced mCRPC but also emphasizes the potential risk of SMF as a serious complication. Although this treatment significantly reduced PSA levels and alleviated symptoms, the occurrence of SMF reminds us of the importance of long-term monitoring for hematological toxicity. Clinicians should be vigilant about this adverse reaction, especially in patients receiving multiple cycles of radionuclide therapy, and adjust the treatment plan accordingly to balance efficacy and safety.

## Data Availability

The original contributions presented in the study are included in the article/Supplementary material, further inquiries can be directed to the corresponding author.
