# Supplementary figures and images for: Actinium-225-PSMA-617 treatment in a patient with advanced prostate cancer causes secondary myelofibrosis: a case report and literature review
Source: Front Med (Lausanne). 2025 Jul 16;12:1569143. doi: 10.3389/fmed.2025.1569143 (PMC12307294; doi:10.3389/fmed.2025.1569143)

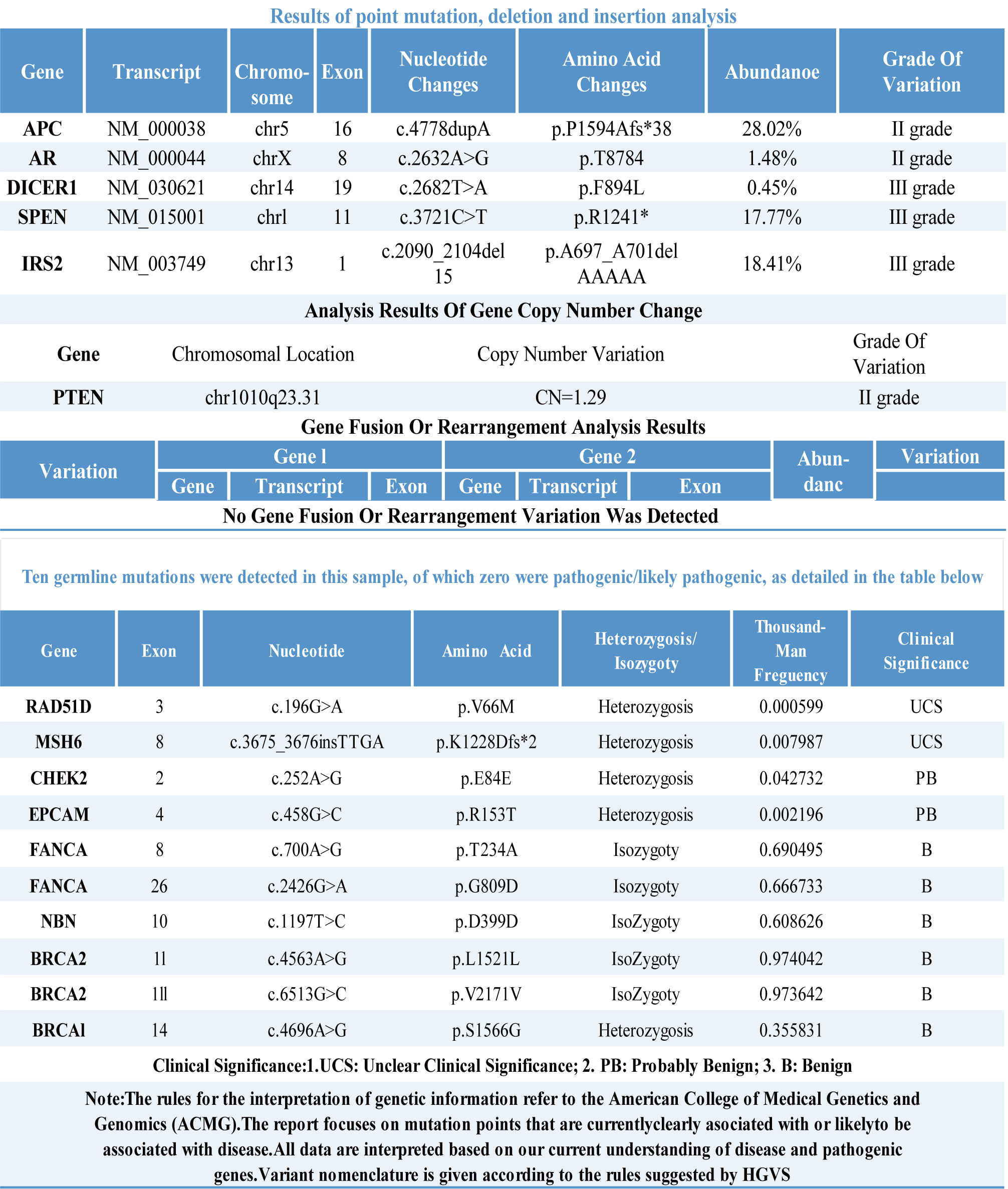

Supplement: Supplementary file 1 [file Image_1.tif]
